# High performance older adults in a population-based sample with low education: Pietà study

**DOI:** 10.1055/s-0042-1759760

**Published:** 2023-03-22

**Authors:** Karoline Carvalho Carmona, Elisa de Paula França Resende, Henrique Cerqueira Guimarães, Thais Helena Machado, Viviane Amaral-Carvalho, Etelvina Lucas dos Santos, Maira Tonidandel Barbosa, Paulo Caramelli

**Affiliations:** 1 Universidade Federal de Minas Gerais, Departamento de Clínica Médica, Faculdade de Medicina, Grupo de Pesquisa em Neurologia Comportamental e Cognitiva, Belo Horizonte MG, Brazil.; 2 Faculdade de Ciências Médicas de Minas Gerais, Departamento de Clínica Médica, Belo Horizonte MG, Brazil.

**Keywords:** Aging, Memory, Healthy Aging, Depression, Aged, Educational Status, Envelhecimento, Memória, Envelhecimento Saudável, Depressão, Idoso, Escolaridade

## Abstract

**Background** Healthy brain aging can be defined as aging without neurological or psychiatric disorders, sustaining functional independence. In addition to the absence of disease and preserved functionality, there are individuals who stand out for their superior performance to that considered normal for their age in cognitive tests. These individuals are called “high-performance older adults” (HPOA).

**Objectives** To investigate the presence of HPOA in an oldest-old population with low education, and if present, to investigate associations with sociodemographic, clinical, and lifestyle variables.

**Methods** We evaluated 132 cognitively healthy individuals from the Pietà Study, a population-based investigation with 639 participants. We used the delayed recall from the Rey Auditory-Verbal Learning Test to verify the existence of HPOA and to classify participants based on their performance. Sociodemographic, clinical, and lifestyle variables associated with HPOA were investigated.

**Results** We identified 18 individuals fulfilling HPOA criteria (age: 77.4 ± 2.6 years old; 14 women; education: 4.6 ± 3.4 years). The other participants, 114 total (age: 79.8 ± 4.5 years old; 69 women; education: 3.0 ± 2.7 years) were classified as “standard performance older adults” (SPOA). In multivariate analysis, younger age (odds ratio [OR] = 0.672; 95% confidence interval [CI]: 0.462–0.979; *p* = 0.037) and lower scores on the Geriatric Depression Scale (OR = 0.831; 95%CI: 0.688–0.989; *p* = 0.038) were associated with HPOA.

**Conclusions** The present study identifies for the first time HPOA with low educational level, thereby reinforcing the existence of biological substrates related to this condition. Furthermore, the data suggest an association between younger age and less depressive symptoms with HPOA.

## Resumo

**Antecedentes** Envelhecimento cerebral saudável pode ser definido como envelhecimento sem transtornos neurológicos ou psiquiátricos e com independência funcional. Além da ausência de doença e funcionalidade preservada, existem indivíduos que se destacam pelo desempenho superior ao normal em testes cognitivos. Estes indivíduos são chamados de “high performance older adults” (HPOA, na sigla em inglês).

**Objetivos** Investigar a presença de HPOA em uma população de idosos com baixa escolaridade e, se presente, investigar associações com variáveis sociodemográficas, clínicas e de estilo de vida.

**Métodos** Avaliamos 132 indivíduos cognitivamente saudáveis do Estudo Pietà ( *n =* 639). Foi utilizado o Teste de Aprendizagem Auditivo-Verbal de Rey para verificar a existência de HPOA e classificar os participantes em dois grupos com base em seu desempenho. Variáveis sociodemográficas, clínicas e de estilo de vida associadas a HPOA foram investigadas.

**Resultados** Identificamos 18 indivíduos que preencheram critérios para HPOA (idade: 77,4 ± 2,6 anos; 14 mulheres; escolaridade: 4,6 ± 3,4 anos). Os demais, 114 no total (idade: 79,8 ± 4,5 anos; 69 mulheres; escolaridade: 3,0 ± 2,7 anos), foram classificados como “standard performance older adults” (SPOA, na sigla em inglês). Na análise multivariada, menor idade (odds ratio [OR] = 0,672; intervalo de confiança [IC] 95%: 0,462–0,979; *p* = 0,037) e menor pontuação na Escala de Depressão Geriátrica (OR = 0,831; IC95%: 0,688–0,989; *p* = 0,038) foram associados ao grupo HPOA.

**Conclusões** O presente estudo identifica pela primeira vez HPOA entre indivíduos de baixa escolaridade, reforçando a existência de substratos biológicos relacionados a esta condição. Além disso, os dados sugerem uma associação entre idade mais jovem e menos sintomas depressivos com HPOA.

## INTRODUCTION

Healthy brain aging can be defined as aging without any neurological or psychiatric disorders and functional independence. In addition to the absence of disease and preserved functionality, there are individuals who stand out for their superior performance to that considered normal for their age in cognitive tests. These individuals are called “SuperAgers” by some research groups, 1 2 or “high-performance older adults” (HPOAs) in a broader definition that takes into account individuals from low- and middle-income countries (LMIC). 3

High-performance older adults are defined according to age and cognitive criteria, while considering the differences between high-income countries and LMIC, and the limitations regarding the normative values of cognitive tests in individuals with low education. 3 Therefore, age ≥ 75 years old and scores in cognitive tests greater than or equal to the scores of younger individuals (50 to 65 years old) define HPOA in LMIC. 3

To date, there is no consistent evidence about the factors that make the cognitive performance of these subject exceptional. One of working hypotheses is the participation of biological factors. A systematic review showing structural and molecular brain preservation of HPOA 4 corroborates this hypothesis. This review included the studies of The Northwestern University Superaging Project. Their structural analyses showed that these subjects do not have expected age-related atrophic patterns. 1 5 On the contrary, they have higher cortical thickness of the anterior cingulate cortex compared with the same age group with average memory performance. 1 5 Histopathological data from the same study has shown that “SuperAgers” have lower density of Alzheimer disease pathological markers, with less neurofibrillary degeneration in the region of the anterior cingulate. 6 On the other hand, they also have greater amount of Von Economo neurons in the same region. 6 A lower frequency of the ɛ4 allele of the *Apolipoprotein E* gene was also found in these high cognitive performance subjects, compared to age-matched adults with usual aging. 1

Another perspective concerns the potential role of environmental factors such as education and social networks in cognitive preservation. A study on SuperAgers found that they had greater levels of positive relations with others than their cognitively average peers. 7 In Latin America, an Argentine study did not find differences between SuperAgers and subjects with normal aging concerning environmental factors, including years of schooling. 8

Although education is often associated with cognitive reserve and brain maintenance, participants with high cognitive performance from previous studies were highly educated, even in the few investigations from LMIC.

Thus, the presence of HPOA in populations with very low levels of education has not yet been described. Furthermore, we still do not have sufficient studies investigating the HPOA in LMIC. The purpose of our study was to investigate the existence of HPOA in a community sample of oldest-old and low-educated individuals, and if so, to investigate sociodemographic, clinical, and quality of life variables possibly associated to HPOA. We hypothesized that there are HPOAs with low education, based on studies that point to the existence of biological determinants for these subjects.

## METHODS

### Participants

Participants were selected from the Pietà study, a population-based investigation on brain aging conducted in Caeté, state of Minas Gerais, Brazil. 9 Drawing on census records, investigators invited all residents living in urban or rural areas who were ≥ 75 years old ( *n* = 1,251) to participate, of whom 51.1% ( *n* = 639) accepted and were fully evaluated.

The participants answered a detailed and structured questionnaire including: identification and sociodemographic information, socioeconomic level (from A = higher to E = lower), 10 quality of life evaluation (World Health Organization Quality of Life - Older adults module [WHOQOL-OLD] protocol) 11 global functional information, 12 mobility, current and previous physical activity, leisure activities (reading habits, games, manual work, participation in social groups and meetings) both currently and in the past, information on religious beliefs and attendance of religious cults (currently and in the past), smoking and drinking habits (currently and in the past; including the four questions from the Cutting Down, Annoyance, Guilt, Eye-Openers (CAGE) Questionnaire, 13 sleep habits, nutritional information (semiquantitative assessment of weekly consumption frequency of meat, carbohydrates, fruits, vegetables, cereals, dairy products, candies and coffee), and self-reported information on hearing and visual function.

They were also submitted to a second structured evaluation that included: brief clinical history (focusing on neurological and psychiatric symptoms), previous and current medical diagnoses and additional medical information (current medications in use with specific dose regimens, frequency of attendance to medical consultation or hospitalizations in the previous 12 months, previous surgeries, falls or fractures).

They underwent thorough physical, neurological, and psychiatric evaluations to determine their clinical, functional, and cognitive status. The clinical evaluation comprised general physical examination (weight and height, blood pressure and heart rate assessment, cardiac and cervical auscultation) and measurement of the brachial-ankle index (using a doppler device). The neurological examination was systematized, with emphasis on motor aspects, and incorporated the motor section of the unified Parkinson disease rating scale (United Parkinson Disease Rating Scale motor [UPDRSm]). 14 The cognitive assessment consisted of the Mini-Mental State Examination (MMSE) 15 and the Brief Cognitive Screening Battery (BCSB), 16 which includes a visual memory test, category fluency test (animals per minute) and clock-drawing test. Functional evaluation consisted of the Pfeffer Functional Activities Questionnaire (QAF) 17 and the Functional Assessment Scale for Alzheimer disease (FAST). 18 The 15-item Geriatric Depression Scale (GDS-15) 19 and the Mini International Neuropsychiatric interview (M.I.N.I) 20 were used to investigate psychiatric disorders. All participants with suspected cognitive impairment and a random subset of cognitively healthy individuals were referred to a thorough neuropsychological assessment, which consisted of Mattis Dementia Rating Scale (DRS), 21 Clinical Dementia Rating (CDR) Scale, 22 naming and praxis tests from the Consortium to Establish a Registry for Alzheimer Disease (CERAD), 23 phonemic verbal fluency task – (Frontal Assessment Battery [FAS]), , 24 and the Rey Auditory-Verbal Learning Test (RAVLT). 25 Further details on the characterization of this sample have been provided elsewhere. 9 The study was approved by the Ethics Committee of the Universidade Federal de Minas Gerais and all participants or their legally acceptable representatives signed the written informed consent.

For the present study, we evaluated a subsample of 132 cognitively healthy individuals defined as absence of any neurological or psychiatric disturbance and on presence of functional independence, based on clinical, neuropsychological, and functional evaluations, in whom the RAVLT test was available. We used the RAVLT delayed recall task (RAVLT A7) to verify the presence of individuals with high memory performance in this sample. The cutoff point used in this task was nine, the same as in The Northwestern University Superaging Project, 1 which means a performance equal to or greater than that of individuals aged between 50 and 60 years old, with higher levels of education. A Brazilian normative study, also with higher education, found similar values for the RAVLT A7 for the age group between 50 and 64 years old. 26

The subjects were divided into two groups: Standard performance older adults (SPOAs) and HPOAs. The participants who scored less than nine in the RAVLT A7 were classified as SPOA. The participants who scored nine or more in the RAVLT A7 were classified as HPOA. In addition, we certified that all participants performed above cutoff points adjusted for education in the MMSE and BCSB visual memory and category fluency tests. Moreover, they did not show any impairment in the neuropsychological assessment, including the DRS. 9

### Statistical analysis

Data were analyzed using PASW Statistics for Windows version 18 (SPSS Inc, Chicago, IL, USA). 27 Normal distribution of numerical variables was verified by the Shapiro-Wilk test. The Student *t* test was used for normally distributed variables in the univariate analysis, while the Mann-Whitney test was performed for parametric variables with non-normal distributions. The categorical variables were compared using the Pearson x ^2^ test or the Fisher exact test.

The significance level of *p* ≤ 0.15 was used to select the variables from the univariate to the multivariate analysis. Effect-size measurement was also performed. Common language effect-size was used for numerical variables, while Phi (2 × 2 table) or Crámer (rxs table, where r or s > 2) was used as an effect-size measure for categorical variables.

In the multivariate analysis, a binary logistic regression model was adjusted to estimate the odds ratio (OR) of the significant variables and other variables known to be associated with cognitive performance ( Table 1 ). The backward selection was applied to identify the final model, with *p* < 0.05 as the selection threshold to retain variables.

**Table 1:** Multivariate analysis

Significant variables	Other variables included
Age	Marital status
Education	Profession
Retired	Present physical activity
Number of meals eaten out	Present reading habits
Past physical activity	Past reading habits
Number of falls in the last year	Participation in social meetings (present)
Weight	Participation in social meetings (past)
Systolic blood pressure	Alcohol consumption (present)
Cardiac rate	Alcohol consumption (past)
UPDRSm total score	Brachial ankle index
Bradykinesia (UPDRS subitem)	Body mass index
GDS total score	Diastolic blood pressure
Fear (GDS subitem)	The Other GDS sub-items
Feeling of empty life (GDS subitem)	Hours of sleep during night
Feeling of inferiority (GDS subitem)	Hours of sleep during day
Abandonment of interests (GDS subitem)	Sleep satisfaction
F26.1 (WHOQOL-OLD)	
F27.3 (WHOQOL-OLD)	
F29.3 (WHOQOL-OLD)	

Abbreviations: GDS, Geriatric Depression Scale; UPDRSm, United Parkinson's Disease Ratio Scale (motor section); WHOQOL-OLD, World Health Organization Quality of Life – Old Module.

## RESULTS

**Figure 1:**
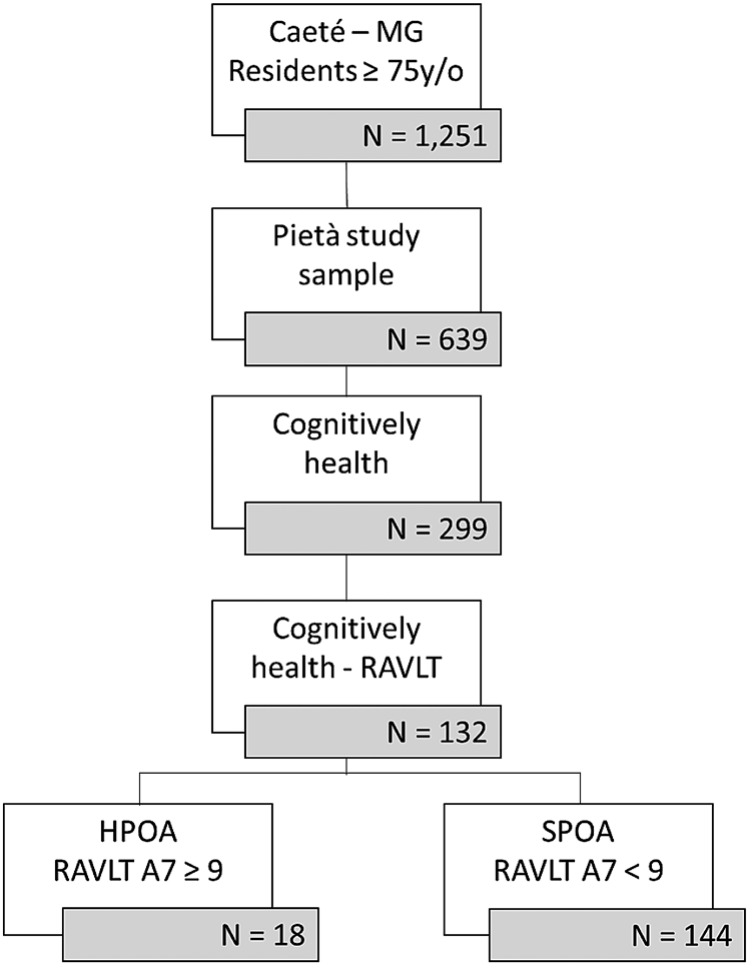
Flowchart for the selection of participants.

Of the 639 participants from the original sample, 299 were cognitively healthy (that is, had no cognitive impairment). Of these, 132 underwent RAVLT and were categorized into SPOA and HPOA ( Figure 1 ).

The sample of cognitively healthy individuals who underwent RAVLT ( *n* = 132) comprised mostly women (63%), with a mean age of 79.5 ± 4.4 years old and mean education of 3.3 ± 2.8 years, including 22 illiterate individuals. We found 18 HPOA, 14 women and 4 men, with a mean age of 77.4 ± 2.6 years old and mean schooling of 4.6 ± 3.4 years, including two illiterate individuals. The SPOA comprised 69 women and 45 men, with a mean age of 79.8 ± 4.5 years old and mean schooling of 3.0 ± 2.7 years.

Compared to the SPOA group, the HPOA group was younger, had a higher educational level and a better global cognitive performance assessed by the DRS. They also had fewer depressive symptoms assessed by the GDS-15, as well as less Parkinsonian signs as assessed by the UPDRSm. The FAQ shows that both groups were completely independent for activities of daily living (ADL). The sociodemographic and clinical characteristics as well as the cognitive and functional assessments of each group are depicted in Table 2 .

**Table 2:** Sociodemographic and clinical characteristics of the study participants

Variables	SPOA	HPOA	Total	*p-value*
*n*	114	18	132	
Sex (Female/Male)	69F/45M	15F/3M	83F/49M	0.172
Age (years ± SD)	79.8 ± 4.5	77.4 ± 2.6	79.5 ± 4.4	0.039
Education (years ± SD)	3.0 ± 2.7	4.6 ± 3.4	3.3 ± 2.8	0.031
Hypertension (Frequency %)	88 (72.2)	16 (88.9)	104 (78.8)	0.212
Diabetes (Frequency%)	13 (11.4)	3 (16.7)	16 (12.1)	0.376
MMSE Median (1 ^st^ –3 ^rd^ quartile)	24.5 (22.0–26.0)	25.5 (22.8–28.0)	25.0 (22.0–26.8)	0.116
DRS Median (1 ^st^ –3 ^rd^ quartile)	123.5 (110.0–132.0)	135.0 (125.3–139.3)	124.5 (111.3–134.0)	0.004
RAVLT A1 Median (1 ^st^ –3 ^rd^ quartile)	4.0 (3.0–4.0)	5.0 (4.0–6.0)	4.0 (3.0–5.0)	0.001
RAVLT A2 Median (1 ^st^ –3 ^rd^ quartile)	6.0 (5.0–7.0)	7.0 (6.7–8.2)	5.0 (5.0–6.0)	< 0.001
RAVLT A3 Median (1 ^st^ –3 ^rd^ quartile)	7.0 (5.0–8.0)	9.0 (8.0–10.0)	6.0 (5.0–8.0)	< 0.001
RAVLT A4 Median (1 ^st^ –3 ^rd^ quartile)	7.0 (5.2–9.0)	10.5 (9.0–12.0)	7.0 (5.0–9.0)	< 0.001
RAVLT A5 Median (1 ^st^ –3 ^rd^ quartile)	8.0 (6.0–10.0)	11.5 (10.0–13.0)	8.0 (6.0–9.0)	< 0.001
RAVLT A7 Median ((1 ^st^ –3 ^rd^ quartile)	6.0 (3.0–7.7)	10.0 (9.0–10.0)	5.0 (2.0–6.0)	< 0.001
FAQ Median (1 ^st^ –3 ^rd^ quartile)	0.0 (0.0–1.0)	0.0 (0.0–0.0)	0.0 (0.0–1.0)	0.326
GDS Median (1 ^st^ –3 ^rd^ quartile)	2.0 (1.0–3.0)	1.0 (1.0–2.0)	2.0 (1.0–3.0)	0.030
UPDRSm Median (1 ^st^ –3 ^rd^ quartile)	2.0 (1.0–5.0)	0.0 (0.0–2.0)	2.0 (0.0–5.0)	0.008

Abbreviations: FAQ, Functional Activities Questionnaire; GDS, Geriatric Dementia Scale; HPOA, High Performance Older Adults; Mattis DRS, Mattis Dementia Rating Scale; MMSE, Mini-Mental State Examination; RAVLT, Rey Auditory-Verbal Learning Test; SPOA, Standard Performance Older Adults; UPDRSm, Unified Parkinson's Disease Rating Scale motor.

In the final model of the multivariate analysis, the variables age and the GDS-15 total score remained significant ( Table 3 ). Age was inversely and significantly associated with HPOA. The decrease of 1 year of age was associated with a 32.8% (1-OR) greater chance of belonging to the HPOA group (95% confidence interval [CI]: 2.1–53.8%). The GDS total score was also inversely associated with HPOA. The decrease of 1 unit in the GDS total score was associated with a 16.9% (1-OR) greater chance of belonging to the HPOA group (95%CI: 1.1–30.8%).

**Table 3:** Logistic regression model

Variable	Coefficient	OR (95%CI)	*p-value*
Age	−0.185	0.672 (0.462–0.979)	0.037
GDS-15 total score	−0.397	0.831 (0.698–0.989)	0.038

Abbreviations: GDS-15, Geariatric Dementia Scale; OR, odds ratio.

Note: Hosmer-Lemeshow: 0.707; R ^2^ Nagelkerke: 0.152.

Among the GDS-15 depressive symptoms, feelings of empty life, inferiority, fear, and abandonment of interests showed a significant association with the outcomes in the univariate analysis.

The adjusted model proved to be adequate, as it presented a Hosmer Lemeshow value of 0.707. This model has a Nagelkerke R2 coefficient of 0.152, indicating that the variables age and GDS-15 total score explain 15.2% of the outcome variability.

## DISCUSSION

In the present community-based sample of low-educated individuals aged ≥ 75 years old, we found 18 participants who presented with exceptional episodic memory. We evaluated clinical, sociodemographic and lifestyle variables possibly associated with HPOA, comparing a group of oldest-old individuals with this profile with a group classified as SPOA. Despite the large number of variables analyzed, HPOA individuals differed from SPOA individuals only on the fact they were younger and had fewer depressive symptoms assessed by the GDS-15. The other analyzed variables were not significant.

Most studies on HPOA/SuperAging have included highly educated participants. The Northwestern SuperAging Program has participants with 15 to 17 years of mean schooling. 1 Thereby, more than the significant associations found in our study, the most interesting finding was to identify HPOA individuals in a sample with very low educational level, including two illiterate individuals.

We know that early-life education is a potentially modifiable risk factor for dementia and that years of education can translate into better cognitive reserve. 28 Although we recognized that high educational level contributes to a better performance in cognitive tests (including the RAVLT, that is known to be strongly influenced by schooling) 26 and to cognitive reserve, the results of the present study suggest that brain maintenance plays an important role in these individuals and its biologicals substrates are probably major contributors to this phenotype. These results reinforce the participation of biological determinants in HPOA, corroborating the notion that SuperAging may have a biological signature.

The expected association between HPOA and younger age have already been shown in previous studies. In the Health ABC Study, younger age was associated with maintaining high performance in the MMSE during 8 years of follow-up. 29 Goveas et al. 30 found an association between younger age and better cognitive performance in a group of 2,228 older women. In our sample, the increase of 1 year of age meant that the individual had a 32.8% lower chance of belonging to the HPOA group.

The other association found in our study relates to the total score on the GDS-15, which was significantly lower in the HPOA group. Depressive symptoms of fear, inferiority, empty life, and abandonment of interests were the contributors most associated with this difference. Some studies have already shown association of less depressive symptoms and a greater feeling of well-being with better cognition. Goveas et al. 30 found a lower frequency of depressive symptoms, assessed by GDS-15, as a predictor of better cognitive outcome. In another study, people ≥ 75 years old without depressive symptoms had better scores on cognitive tests than older adults with depressive symptoms. 31 On the other hand, depressive symptoms have always been closely associated with cognitive complaints and cognitive impairment. This topic is often controversial due to the discussion about whether depressive symptoms are responsible for cognitive decline or are part of the dementia process, as an initial symptom. Nonetheless, several studies point to the association between depressive symptoms and worse cognitive performance, showing ∼ 50% of older adults with depressive disorder having cognitive symptoms. 31 32 Therefore, the association between depressive and cognitive symptoms in older adults is already well studied and established, while the association between absence of depressive symptoms and HPOA needs further investigation and better understanding.

Some limitations of our study should be mentioned. First, the cross-sectional nature of the study does not allow drawing conclusions on causality. Second, the homogeneous sample from the same region and with similar habits and customs may limit the application of the results to a more heterogeneous group. Third, as we do not have enough literature data about variables related to HPOA, particularly in low-educated populations, we had to resort to backward selection, an automatic selection method, in the statistical analysis. Lastly, the scarcity of validated memory tests for low-educated subjects and a lack of neuropsychological assessment tools better adapted for oldest-old individuals may have underestimated the number of participants in the HPOA group.

In summary, our results show that HPOA may occur even among very low-educated elderly and that it is associated with younger age and fewer depressive symptoms. We believe our study contributes to the field of understanding HPOA in the context of low educational level and to a better characterization of oldest-old individuals, which is the fastest growing segment among the older adult population.
